# The Long Shadow of Fatalism: a Philosophical Speculation on Forster’s “the Machine Stops” (1909) on the Disintegration of Technologically Advanced Societies Back Then and Today

**DOI:** 10.1007/s40926-021-00165-1

**Published:** 2021-02-10

**Authors:** Peter Seele

**Affiliations:** USI Lugano, Via Buffi 13, CH-6900 Lugano, Switzerland

**Keywords:** Philosophy of literature, Ethics, Futurism, Fatalism

## Abstract

EM Forster’s short story “The Machine Stops” from 1909 is widely reread and discussed again for some ten years as it portrays a science-fiction world resting on similar technological advancements as today in the digital era. Also management literature reviewed the short story with regard to centralized decision making, rationality and totalitarianism. I argue instead, that the main theme of the short story is – in Forster’s own words – the closing of a civilization in times of transition and facing major challenges. I built the argument by original quotes from Forster and by portraying the years 1906–9, when Forster developed the short story. This era before the Great War starting in 1914 was characterized by euphoric ‘futurism’ based on groundbreaking innovations like ‘long distance messaging’, ‘penny post’, ‘animated films’, Ford’s assembly line, ‘Olivetti typewriter’, ‘feature film’, ‘large ships’ and ‘air transportation’ – the ingredients of the short story as I argue. At the same time these acquitted years were characterized by increasing disintegration, instability, rebellions and a financial crisis with bailout programs. Based on the analogy and as part of speculative philosophy I reconstruct the current great challenges with Forster’ shadow of fatalism and arrive at the urgency to put more effort in addressing and researching pathways out of the crisis and towards stabilization of business and society.

## Starting Point: The Two Themes of ”The Machine Stops”

At first sight, the science fiction short story “The Machine Stops” (TMS) from 1909 from Edward Morgan Forster anticipates technological innovations like the internet, social media, video telephones and the compartmentalization of society a century before they became reality. This visionary power of the short story has led to a noteworthy relaunch and reception of the book in recent years. A recent management theory paper on algorithmic decision making is building on “The Machine Stops” (Lindebaum et al. 2019) and is in line with reviews (Alfred 2010; Thompson 2018) of the relaunch of Forster’s short story in the recent years linking the short story to recent developments of the digital transformation and how it impacts business and society at large. The contribution for management theory from this piece of literature is anchored in decision making, more specifically on algorithmic decision making seen though a Weberian perspective of rationality.

I argue – with Forster himself and e.g. Thompson (2018) – that algorithmic decision making as main line of reception is not the main argument of the short story, but more of an emergent sub-theme enabled in recent years by the visionary power of Forster – and the technological advancements of our time. In other words: Framing TMS with computers and algorithms is not originally based on Forster, as only some of the features like video-calls and the name of ‘the machine’ for a totalitarian system in a technologically advanced world appear to be familiar to our world and technology, based on software computing and executed by algorithms. In sum: ‘the machine’ as portrayed by Foster says nothing about algorithms as we know them today. Forster describes living in ‘the machine’ in a way that may also be entirely analogue (“*pneumatic post*”). Instead – as the title of the short story already suggests – the core argument is the disintegration of a technologically advanced and enhanced society: “The Machine Stops”. The term “machine” may also be seen as a *pars-pro-toto* science fiction concept of the dissolving society in the times before the Great War, similar to the atmosphere described in Thomas Mann’s description of pre-war Europe in “The Magic Mountain” (“Der Zauberberg”, writing started in 1912) or Herrmann Hesse’s “Demian” (Hesse 1919), just to mention two Nobel prize awarded fiction authors, who began their work on the texts also before the Great War started in 1914.

In this text however I do not want to disprove the interpretations of Lindebaum et al. (2019), as their theme and reflection of rationality stands for itself and builds on a rich body of literature also in the philosophy of management (Hartman 2015). Instead I want to present the reception of “The Machine Stops” in our time and add the inconvenient and maybe fatalistic point, that the core argument is disintegration of society, where advanced but dysfunctional technology acts as a catalyst. And as a contribution in speculative philosophy I want to extrapolate, that there are structural analogies between pre-war Europe and (potentially or perceived by some) today with major pillars of the current open societies under fire, such as disinformation, financial instability, geopolitical shifts and last but not least the environmental crisis. Based on this expanded reading of “The Machine Stops” this text adds to the theoretical implications not only regarding digital technology, but also in a more holistic perspective incorporating the crisis phenomena of our times, possibly fueling disintegration of our current global state of affairs, advancing and reinforcing the urgency of ethical and sustainable measures for business and society.

## Technological and Political Disruptions 1906–1909 in the Years of ‘Futurism’

In the following I support the argument, that the core theme of “The Machine Stops” is about “civilization’s long day was closing” (from Forster 1909), a symbolic and atmospheric description of pre-war Europe in times of turbulences and an atmosphere of enthusiastic, speed-loving ‘futurism’ (Marinetti 1909). The original short story was written and published first in 1909, five years before the Great War started. Therefore, a closer look into the time of the conceptualization and write up of “The Machine Stops” may shed some light on both the anticipation of advanced digital technology (as we have it today*) and* the disintegration of pre-war Europe. Given that in “The Machine Stops” global travelling, real time video telephony and exchange via social media is anticipated (without going into details how it is technically organized), a selective enumeration of the innovations and events on the two levels of technology and disintegration from the three years preceding the short story summing up in the Great War is given.

In addition 1909 also was the year, when the “Manifesto of Futurism” was written by Filippo Tommaso Marinetti (1909) emphasizing technology, industrial cities and ‘fast’ objects such as cars and airplanes. Marinetti recently was revisited discussing the link between futurism and how it led to fascism and why it could happen again with current techno utopianism (Eveleth 2019). So here is a closer look at the years, when Foster developed and wrote the short story. The following Table 1 is – unlike in a systematic literature review – no comprehensive or systematic list of events and innovations, but a compilation after reading various history books and encyclopedias, a ‘method’ that may be described as ‘snowball reading’ in analogy to ‘snowball sampling’ and in collecting interview data. Combining Technology with disintegration and instability however is a link based on the subjective correlation as part of speculative philosophy and does not follow a causal relation or not even statistical correlation.

**Table 1:** Selected innovations in pre-war Europe 1906–1909, when Forster developed ‘The Machine Stops’

Year	Technology and Globalization	Disintegration and Instability
1906	RMS Lusitania is launched, the world’s largest ship U-1: First imperial German Navy Submarine First officially reported powered flight in Europe in Bagatelle, France Gandhi coins the term Satyagraha (non-violence) SOS becomes an international distress signal First feature film: The Story of the Kelly Gang Austin car model ‘15/20’ and ‘25/30)	Persian constitutional revolution Pope Pius X denounces the French law on the separation of Churches and the State Bambatha Rebellion of the Zulu against British rule and taxation First State Duma of the Russian Empire Second Occupation of Cuba by the U.S. to control a rebellion
1907	The first taxi cab with taximeters begin operating in London UPS is founded in Seattle The triode thermionic amplifier is invented and starts the development of electronics Autochrome Lumiere is the first commercial color photography process. Porsche car model ‘Maja’	Within less than 2 weeks 5 shops are wrecked, hundreds of lives are lost The Romanian-Peasants’ Revolt results in possibly as many as 11,000 deaths The Second Hague Peace Conference opens Major financial crisis is averted when Wall-Street financiers create a 25,000,000 pool to invest in the shares on the plunging NYSE, ending the panic of 1907 In Chile over 2000 workers are killed by soldiers firing at striking mine workers
1908	A long-distance radio-message is sent from the Eiffel Tower for the first time The first around-the-world car race begins Henri Farman made the world’s first flight with a passenger The Hoover Company acquired manufacturing rights to the portable vacuum cleaner just invented Emile Cohl makes the first fully animated film Henry Ford produces his first Motel T Penny Post between the U.K. and the U.S. Olivetti (typewriter) is founded in Italy Foundation: Bureau of Investigation in U.S., now FBI Fiat car model ‘1’	Ferdinand I of Bulgaria declared himself ‘Tsar of Bulgaria’ and strived for a “new Byzantium” leading to the Balkan Wars. The Bosnian crisis begins, after the Austro-Hungarian Empire annexes Bosnia and Herzegovina Tweedmouth-Affair triggering the Dreadnought-Program and rigging of the British and German navy Daily-Telegraph-Affair worsening the German-British relations.
1909	British Nimrod Expedition to the South Pole Anglo-Persian Oil Company (today BP) is incorporated The U.S. army purchases the world’s first military airplane Cadillac model ‘Thirty’	Serbia accepts Austrian control over Bosnia and Herzegovina. Tragic Week: Barcelona experiences a workers’ uprising In Nicaragua, 500 revolutionaries (including 2 Americans) are executed and the U.S. responds by sending 2 warships

One could argue that every year innovations are created and conflicts touch surface. However, if we connect some of the dots in pre-war Europe mentioned in this selection, we get the impression that Forster extrapolated some of the inventions to ‘invent’ the ‘machine’ for his short story, described by historians today as ‘mediatization of the air’ from 1900 to 1910 (Rikitianskaia et al. 2018).

Here is the ‘train of thoughts’ from the above Table 1: Take the first “color photography” plus the first ‘long distance messaging’ plus ‘penny post’ plus ‘animated films’ plus Ford’s assembly line plus ‘Olivetti typewriter’ plus ‘feature film’, mix it, shake it well and recombine the elements in a fantastic, science-fiction way, you might indeed end up with video telephony, accelerated messaging leading to proto-social media and addictive communication, all coordinated by an all encompassing system called ‘the machine’, that can be portrayed as “algorithmic decision-making”, when understanding algorithms also in a non-digital way.

**Fig. 1:**
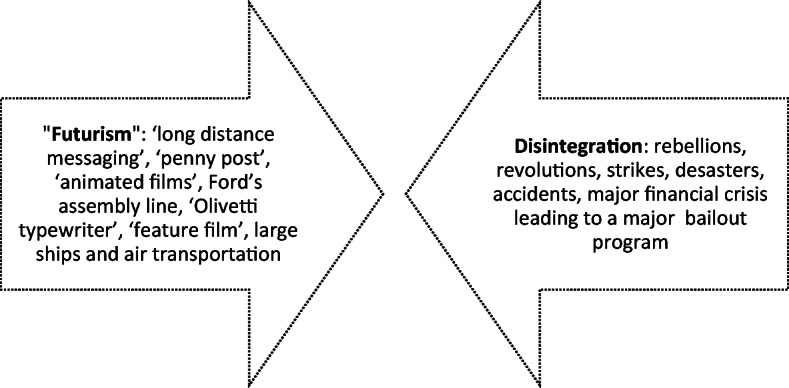
“The Machine Stops” as convergence of pre-war ‘futurism’ and ‘desintegration’ from 1906 to 1909

But next to the technological advancements, there is a second layer: At the same time the years before writing and publishing “The Machine Stops” were characterized not only by ground-breaking technological innovations and an atmosphere of ‘futurism’, but also by disruptions of the existing power-structures, leading to disintegration, instability and turmoil. A mix, consisting of rebellions, revolutions, strikes as well as a major financial crisis bringing the stock market to the brink of a collapse, that could only be prevented by a major bailout program. As a third component technologically fueled globalization was underway with large ships and the creation of air transportation of goods and persons Fig. 1.

Forster, I argue, amalgamated these multilevel disruptions in a centralized entity, the ‘machine’, acting as authority in the totalitarian sense. This totalitarian notion of ‘the machine’ is also addressed in the article by Lindebaum et al. (2019), when pointing to Hanna Arendt’s take on totalitarianism. Here I would go one step further in arguing, that this amalgamation of ‘the machine’ has a build-in tendency to fall apart – just as depicted in the short story and as witnessed in the devastating annihilations of the two great wars following the techno-euphoria leading to fascism. In this regard: Italian ‘Futurism’ is seen in a clear line of developing European fascism, born in Italy and executed in Nazi-Germany, as Griffiths outlines in a remarkable essay on political ancestry (Griffiths 2017).

## From ‘Stops’ to ‘Stop’: Zooming in on the *process* from the First “Cracks” to a “Bloody Halt”

The main argument of TMS is about transitory times of thresholding (Erickson 1999). This is in line with some of the more recent reviews of the short story: Thompson (2018) in an article on the political geographies in E.M. Forster’s “The Machine Stops” calls the short story a “grim fable” about “a self-aware technology, that rules the subterranean world, not with an iron fist, but with millions of steel tentacles” (Thompson 2018: 32). In 2010, the journal *Wired* portrayed the relaunch of “The Machine Stops” and pointed not only to the technological prophecy of a “proto-Google”, but to a “futuristic information-oriented society that grinds to a bloody halt, literally” (Alfred 2010).

For the contribution of this essay I want to shift – building on the words from Forster himself – the focus from the ‘bloody halt’, when the machine literally stops, to the process, before it happened. This shift can also be seen in line with the distinction from the theory of institutional change as put forward in new institutional economics (in the line of D.C. North 1990). Kasper and Streit (1999) distinguish between the Big Bang and the Gradualism approach of transitions. By stressing the processual aspect of “the machine *stops*” the point to be made here is the perspective of gradual changes leading to disintegration and instability: As the title “The Machine Stops” grammatically already signals, the ‘stop’ is a process given the ‘s’, grammatically representing the 3rd person singular presence: The machine *starts* ‘stopping’, when the machine starts to become unreliable, instable and dysfunctional, although still operating. Disintegration is a weak indication of a possible cascading effect (Buldyrev et al. 2010) leading to a total halt and catastrophe: This is what happens in the short story “When the Machine Stops”. This is what happened in history in the years when the short story was conceptualized and written. And this is why this essay suggests to consider fatalism to be added to theory building. Forster explains the first indications triggering the process of ‘stopping’ as follows:

*It became difficult to read. A blight entered the atmosphere and dulled its luminosity. […]. The air, too, was foul. Loud were the complaints, impotent the remedies, heroic the tone of the lecturer as he cried: “Courage! courage! What matter so long as the Machine goes on? To it the darkness and the light are one.” And though things improved again after a time, the old brilliancy was never recaptured, and humanity never recovered from its entrance into twilight. There was an hysterical talk of “measures,” of “provisional dictatorship,” […]. But for the most part panic reigned, and men spent their strength praying to their Books, tangible proofs of the Machine’s omnipotence. There were gradations of terror — at times came rumours of hope — the Mending Apparatus was almost mended.*

What we see here is a description for the extension of coming to a halt. The ‘s’ (as in stop*s*) of disintegration, before the machine finally stops moving at all. But Forster tells us clearly when the process of the ongoing disintegration and dysfunctionality ends and the machine stands still and the disaster breaks loose:

*But there came a day when, without the slightest warning, without any previous hint of feebleness, the entire communication-system broke down, all over the world, and the world, as they understood it, ended.*

Lindebaum et al. (2019) argue, that Vashti embodies formal rationality, whereas Kuno represents the capacity for substantive rationality. I would add, that the mother Vasthi and the ‘machine’ represent dysfunctionality and finally discontinuation, whereas Kuno represents the enabling of a vivid, non-technological way out, outside the ‘machine’. We can also say: The ‘machine’ and the dying inmates stand for the time ontological dimension of the eschatological *Alpha-Omega-Point* with a clear-cut beginning and definite shutdown. Kuno on the contrary represents a *creatio-continuo* approach, where transitions are processual and not definite and life ‘finds a way’ in line with nature and ‘unfiltered air’.

In the conclusion I transpose this argument of disintegration of a civilization to the current state of affairs of business and society.

## Philosophical Speculation on Today’s Situation: Permanent Crisis and Disintegration Revisited

“Apocalypse Becomes the New Normal” is the title of an article of Paul Krugman (2020) commenting on the impact of the climate change in Australia and beyond. The paradoxical title captures quite well the synchronicity of ongoing crisis and coming to a halt. Gramsci found a fitting way to describe the threshold of a crisis before the second great war, where he depicts the transitory phase leading to disintegration, that is at center stage for the point of this text as well: “The crisis consists precisely in the fact that the old is dying and the new cannot be born; in this interregnum a great variety of morbid symptoms appear” (Gramsci 1930: 32). In the following I engage with the speculative argument that in TMS we find structural analogies to depict today’s challenges (Sacks 2019).

In a longue durée perspective Marc Chesney, a quantitative finance professor, asks if the Great War may provide analogies to the financial crisis of 2008 and the bailout programs. In his book from 2014 he argues, that the two crisis were based on similar grounds – and he proposes that we today live in “The permanent crisis”. Chesney (2018) puts the argument forward, that the sinking of civilizations takes place in the name of its salvation, a headline also adequate for ‘the machine’ and its manual in the short story. And possibly for today, if we consider the following crisis ingredients and indications for disintegration:

- Climate and Environmental Crisis: scientific evidence for global warming, desertification, wild fires. Problem of tipping point (Lenton et al. 2019)
- Migration and refugee crisis with millions of people suffering (Puma et al. 2018)
- Financial Crisis: Too big to fail problem, global debt, quantitative easing (Chesney 2018)
- Geopolitical disintegration: Crisis of Nato partners, new players like Chinese Belt and Road Initiative (Seele and Helbing 2018)
- Public Administration: overloaded and understaffed governments leading to partial or temporary shutdowns in all categories of countries. (Morello 2018)
- Digitalization, algorithmic automation and artificial intelligence leading to creative destruction, job losses and challenged legal systems and surveillance capitalism. (Vandekerckhove 2019, Seele et al. 2019:)
- Platform companies that do not serve the human user anymore, but treat the human user as a sellable product in “surveillance capitalism” (Zuboff 2019)
- Media and Journalism crisis and disintegration of old business models: disinformation, fake news, alternative facts, social bots, social media nudging and manipulation (Russ-Mohl 2017)
- Finally: With the Covid-19 pandemic in 2020 (Vandekerckhove 2020) the analogy with TMS is even more apparent. Lockdown life (Gompertz 2020) and the social distancing while working from home office (Pettigrew 2020) are thick descriptions of the world in TMS.

Each of the exemplary and non-comprehensive aspects of contemporary destabilization and disintegration poses challenges for societies, organizations and individuals. However, taken together they might lead to the thread of a systemic shutdown as described in ‘The Machine Stops’ and a large-scale-crisis as the Great War a few years after Foster’s short story.

The theme of ‘The Machine Stops’ as “closing” of a civilization is already widely discussed in today’s research landscape framed as “Grand Challenges” (George et al. 2016). Next to the inclusion of fatalism as cry wolf perspective the practical anticipation to (hopefully) prevent system break-down the aspiration to turn the tide before cascading effects happen is to advance ethics, corporate cocial responsibility and sustainability. In this regard much of what we see in the past years of the CSR literature falls into the category of “too little, too late”, which is also the mantra of the Fridays-for-future movement building on the scientific evidence regarding the sustainability crisis. Rhodes and Fleming harshly criticized one of the most influential CSR theories - political CSR – to be abandoned, as it “reflects both a triumph of neoliberal corporate power and a harbinger of democracy’s demise” (Rhodes and Fleming 2020). Therefore future theory building – triggered by fatalism – could overcome the cherry picking practices of e.g. CSR reporting (Milne and Gray 2013) or corporate greenwashing (Seele and Gatti 2017) by a much stronger and transformative concept of responsibility. If the UN’s SDGs are appropriate to bring about the required change is still an open question. Building on the existing literature one might consider – in the light of the possibility of a societal catastrophe like a great war, loss of the biosphere, global pandemic or global economic downturn (or all at the same time) – to integrate the theoretical concept of fatalism into theorizing about management and business. Not to fuel negative consequences, but in order to cope with fatal threats and incidents and to develop anti-apocalyptic strategies, in which organizations play a major role. Fatalistic thinking may not only be seen as psychological perspective as downward spiral. Buldyrev et al. (2010) point to cascades of failures in interdependent networks, where the dynamics towards catastrophic outcomes depends on the interdependence. Fatalism as a cry wolf concept may serve to identify the early indications and may inspire to take measures against interdependencies and towards resilience and stability. Theoretically this could be achieved by expanding the theoretical scope of existing theoretical frameworks on the grand challenges mentioned above by integrating insights from disciplines such as ‘social physics’ (Pentland 2014), ‘complexity science’ (Helbing and Kirman 2013) or ‘computational social science’ (Miller and Page 2007). Practically this could be achieved by a more rigorous digitally enforced and blockchain based supply chain management (Dierksmeier and Seele 2019) or by a global governance standard based on key performance indicators of the United Nations Sustainable Development Goals.

“The Machine Stops” helps us as a warning from the past, that downplaying the signs of destabilizing disruptions is an invitation to work even harder on the solutions of the current challenges.
